# Change in Body Size and Mortality: Results from the Melbourne Collaborative Cohort Study

**DOI:** 10.1371/journal.pone.0099672

**Published:** 2014-07-02

**Authors:** Amalia Karahalios, Julie A. Simpson, Laura Baglietto, Robert J. MacInnis, Allison M. Hodge, Graham G. Giles, Dallas R. English

**Affiliations:** 1 Centre for Epidemiology and Biostatistics, Melbourne School of Population and Global Health, The University of Melbourne, Melbourne, Victoria, Australia; 2 Cancer Epidemiology Centre, Cancer Council Victoria, Melbourne, Victoria, Australia; Kagoshima University Graduate School of Medical and Dental Sciences, Japan

## Abstract

**Background:**

The association between change in weight or body mass index, and mortality is widely reported, however, both measures fail to account for fat distribution. Change in waist circumference, a measure of central adiposity, in relation to mortality has not been studied extensively.

**Methods:**

We investigated the association between mortality and changes in directly measured waist circumference, hips circumference and weight from baseline (1990–1994) to wave 2 (2003–2007) in a prospective cohort study of people aged 40–69 years at baseline. Cox regression, with age as the time metric and follow-up starting at wave 2, adjusted for confounding variables, was used to estimate hazard ratios (HRs) and 95% confidence intervals (CIs) for change in body size in relation to mortality from all causes, cardiovascular disease and cancer.

**Results:**

There were 1465 deaths (109 cancer, 242 cardiovascular disease) identified during an average 7.7 years of follow-up from 21 298 participants. Compared to minimal increase in body size, loss of waist circumference (HR: 1.26; 95% CI: 1.09–1.47), weight (1.80; 1.54–2.11), or hips circumference (1.35; 1.15–1.57) were associated with an increased risk of all-cause mortality, particularly for older adults. Weight loss was associated with cardiovascular disease mortality (2.40; 1.57–3.65) but change in body size was not associated with obesity-related cancer mortality.

**Conclusion:**

This study confirms the association between weight loss and increased mortality from all-causes for older adults. Based on evidence from observational cohort studies, weight stability may be the recommended option for most adults, especially older adults.

## Introduction

Convincing evidence exists for an association between overweight and obesity, measured by weight or Body Mass Index (BMI), and all-cause mortality [1], [2]. Weight and BMI are crude measures of adiposity that do not directly measure body fat distribution, which is especially important for older adults where body fat distribution changes with an increased centralisation of adiposity from the limbs to the trunk while total fat/weight remains constant [3], [4]. Measures of central or abdominal adiposity (e.g. waist circumference (WC)) are more highly correlated with visceral adiposity, which is more strongly associated than BMI with all-cause mortality and with cardiovascular disease, cancer and type 2 diabetes [5]–[8].

Increasingly, investigators have assessed the effect of change in obesity, measured by weight or BMI on mortality. A meta-analysis of 26 cohort studies showed that unintentional weight loss increased the risk of all-cause mortality, whereas intentional weight loss had a small benefit for unhealthy adults but was associated with a marginal increased risk of death for healthy adults [9]. The authors recommended future studies measure more than just weight or BMI in order to account for fat distribution and the ratio of lean body mass to fat. Three studies of change in WC and all-cause or cause specific mortality gave inconsistent results [10]–[12]

We investigated the associations between changes in WC, weight, or hips circumference (HC), and mortality in a prospective cohort study in Melbourne, Australia, in which anthropometric measurements were performed at baseline and approximately 12 years later.

## Methods

The Melbourne Collaborative Cohort Study (MCCS) is a prospective cohort study of 41 514 people (24 469 women) living in Melbourne, and aged between 27 and 77 years at baseline (99.2% were 40 to 69 years). Participants were recruited between 1990 and 1994 and attended clinics where demographic, anthropometric, lifestyle, and dietary information were collected and anthropometric measurements were performed [13]–[15]. A follow-up clinic was conducted between 2003 and 2007 (wave 2) to update baseline information, and repeat anthropometric measurements. Participants gave written consent to participate in the study. The Cancer Council Victoria's Human Research Ethics Committee approved the study protocol.

### Exposure measures

Height was measured at baseline, to 1 mm, using a stadiometer. At both waves, weight was measured to 100 g using a digital electronic scale, and WC and HC were measured to 1 mm using a 2-meter metal anthropometric tape. The WC was measured at the narrowest part of the torso and the HC was measured at the point of maximum circumference over the buttocks. Participants wore light clothing with belts and restricting garments removed. Changes in WC (ΔWC; cm), weight (ΔWeight; kg) and HC (ΔHC; cm) were calculated as the value at baseline (1990–4) subtracted from the value at wave 2 (2003–7).

At baseline and wave 2, structured questionnaires were administered to participants to obtain and update information about country of birth, whether the participant lived alone, highest level of education, physical activity, smoking status, dietary and alcohol intake data [14], [16].

To account for physical activity at both waves of data collection, participants were asked how much time they spent on low, moderate and high levels of physical activity at home and at work. The responses were categorised as: none at all, one to two times per week; and three or more times per week, and were coded as 0, 1.5 and 4, respectively. These scores were then summed to give an overall physical activity score, with high intensity physical activity receiving double the weight of low intensity physical activity and walking. The total physical activity score for each participant was grouped into the following approximate quartiles: 0; >0 and <4; ≥4 and <6; ≥6.

To account for diet and alcohol consumption at baseline and wave 2, a Mediterranean diet score (ordinal scale from 0 (poor diet) to 9 (good diet)), was created at each wave based on the following components of diet and alcohol consumption: high intake of vegetables, fruits and nuts, legumes, fish and seafood, and cereals; low intake of meat and meat products and dairy products; high ratio of monounsaturated to saturated lipids; and moderate intake of ethanol [17].

Residential postcodes at baseline were used to classify participants into quintiles of an area-based measure of socioeconomic status [18]. Smoking status was categorised as lifetime abstainer, quit before baseline, quit between baseline and wave 2, or current smoker at wave 2 (‘cumulative smoking status’).

### Mortality

Vital status was obtained by probabilistic record linkage to the Victorian Registry of Births, Deaths and Marriages, and the National Death Index. High sensitivity and specificity of linkage to the National Death Index has been reported [19]. Participants were identified as having died from cardiovascular disease (CVD) if the primary cause of death had International Classification of Diseases (ICD)-10 codes of I00-I78, or having died from obesity-related cancer if the primary cause of death had ICD-10 code of C15 (oesophagus), C18–C20 (colorectum), C25 (pancreas), C50 (breast), C54 and C55 (endometrial), or C64 (kidney).

### Exclusion criteria

We used complete-case analysis to handle the missing data. We excluded individuals diagnosed with cancer before wave 2, since cancer might cause weight loss and increase the risk of early mortality. Cancer cases were identified by linkage to the population-based Victorian Cancer Registry and to the Australian Cancer Database to identify cases diagnosed in other states of Australia.

Participants with extreme values for the anthropometric variables (values below the 0.5 and above the 99.5 sex-specific percentiles of WC, weight, or HC at baseline, and of ΔWC, Δweight, and ΔHC) and energy intake were also excluded due to potential measurement errors.

### Statistical analysis

The hazard ratios (HRs) for change in body size for all-cause and cause specific mortality were estimated using Cox regression with attained age as the time metric. Follow-up began at the date of their wave 2 measurements and ended at date of death, date left Australia, or 31 December 2012, whichever came first. To estimate HRs for CVD and obesity-related cancer mortality we fitted competing risk models using the data duplication method [20]. Because information on cause of death was available until 31 December 2010, follow-up for these analyses ended on that date.

We used the likelihood ratio test to test the assumption of a (log) linear association between the change in body size measures and mortality by comparing models with categorical variables categorised into quintiles and pseudo-continuous variables (set to the median value in each quintile). The category representing minimal weight gain, without weight loss, was the reference group. Tests based on Schoenfeld residuals and visual inspection of the log of the cumulative hazard showed no evidence that the proportional hazard assumptions were violated.

A causal diagram was used to choose confounding variables; these were: country of birth, sex, baseline body size measurement, quintile of socioeconomic status, cumulative smoking status, and the following lifestyle measures at baseline and wave 2: an indicator variable of whether the participant lived alone, Mediterranean diet score, and physical activity (Figure S1) [21]–[23].

HRs for change in body size and mortality might vary by sex, country of birth, age at baseline, baseline body size and self-reported health status [24]. As well, smoking, length of time after wave 2 and undiagnosed diseases might change body size and increase the risk of mortality [9], [25]. We conducted sensitivity analyses by fitting separate interaction terms for the following variables: (i) sex, (ii) country of birth (participants born in Australia/New Zealand/United Kingdom and Southern Europe), (iii) age at baseline, (iv) baseline value of body size cut off at the sex-specific mean (WC: 94 cm for men and 80 cm for women; weight: 81 kg for men and 68 kg for women; HC: 101 cm for men and 102 cm for women), (v) self-reported health status at wave 2 (i.e. ‘excellent/very good’ and ‘good/fair/poor’), (vi) smoking status (never versus ever smoked), (vii) length of follow-up after wave 2 (≤3 years of follow-up), (vi) previous history of disease (indicator for angina, diabetes or heart attack reported at baseline or wave 2), with our primary exposure of interest, ‘the change in the anthropometric measure’, and tested the interactions with likelihood ratio tests.

Statistical analyses were performed using Stata version 11.2 [26].

## Results

**Figure 1:**
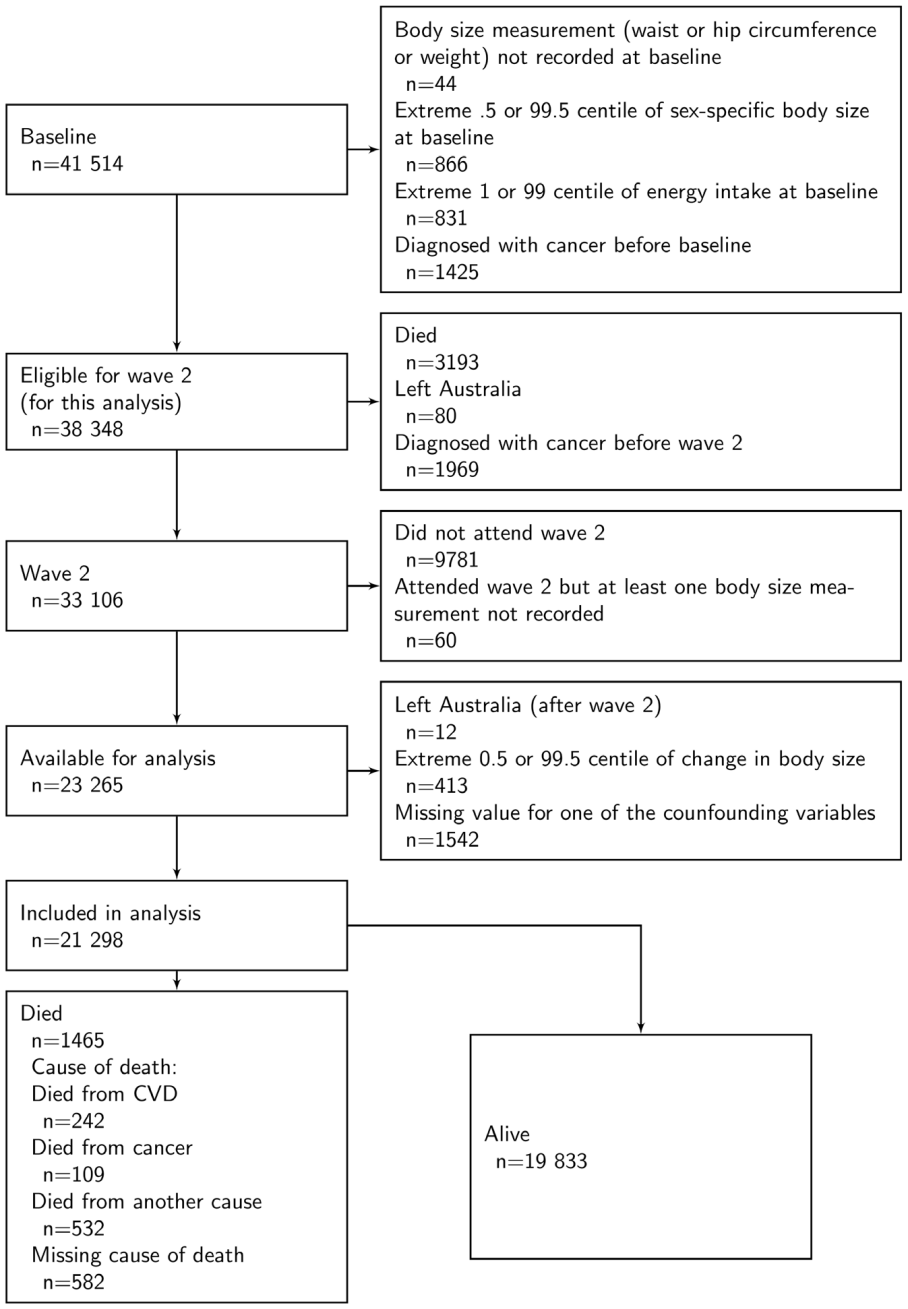
Flowchart of participants in the MCCS.

Of the 41 514 participants, 44 did not have their body size measured at baseline, 866 had a body size measure in the extreme 0.5 or 99.5 sex-specific centile at baseline, 831 had a total energy intake in the 1 or 99 centile at baseline, and 1425 had a diagnosis of cancer before baseline. Between baseline and wave 2, 3273 participants died or left Australia and 1969 were diagnosed with cancer, leaving 33 106 available for invitation to wave 2 and eligible for this analysis. Of these, 9781 (30%) did not attend wave 2, 60 did not have at least one of their body size measurements recorded at wave 2, and 12 left Australia after wave 2. Finally, 1955 were excluded due to missing information for confounding variables at baseline or wave 2, or for an extreme change in body size, leaving 21 298 (13 071 females) with complete data available (Figure 1).

Participants who attended wave 2 were more likely to be born in Australia, New Zealand or the United Kingdom, have a degree or diploma, have low baseline alcohol intake, have never smoked, and be younger (Table S1). The mean WC, weight, and HC at baseline for the participants included in the analysis were 84 cm, 72 kg, and 101 cm, and over an average of 11.8 years between measurements, the mean increases were 7.0 cm, 2.2 kg, and 3.4 cm, respectively (Table 1). About a third (35%) of participants lost weight from baseline to wave 2, whereas only 16% decreased their WC. The body size measurements at baseline and wave 2 were highly correlated (WC, r = 0.82; weight, r = 0.91; HC, r = 0.76; Table S2).

**Table 1:** Distribution of body size measures at baseline and wave 2 for the MCCS participants.

	All	participants	Attended	wave 2	
	Baseline			Baseline	Wave 2
	n	mean (SD)	n	mean (SD)	mean (SD)
**Waist circumference (cm)**					
All	41 514	85.5 (13.0)	21 298	83.5 (12.0)	90.5 (12.5)
Females	24 469	80.0 (11.8)	13 071	78.1 (10.5)	86.1 (11.9)
Males	17 045	93.5 (10.0)	8227	92.1 (8.9)	97.5 (10.0)
**Weight (kg)**					
All	41 514	73.4 (13.7)	21 298	72.3 (12.7)	74.5 (13.6)
Females	24 469	68.2 (12.4)	13 071	67.2 (11.0)	69.7 (12.4)
Males	17 045	80.8 (11.8)	8227	80.4 (10.7)	82.1 (11.9)
**Hip circumference (cm)**					
All	41 514	101.4 (8.9)	21 298	100.6 (7.9)	104.0 (8.9)
Females	24 469	101.6 (10.0)	13 071	100.6 (8.8)	104.4 (10.0)
Males	17 045	101.1 (7.1)	8227	100.5 (6.2)	103.3 (6.6)

### All-cause mortality

There were 1465 deaths (242 CVD deaths and 109 obesity-related cancer deaths of the 883 deaths with primary cause data available) occurring on average 7.7 years after wave 2. Only 12 (0.03%) participants left Australia and only 65 (4%) deaths were attributed to an external cause of death (i.e. an ICD 10 code of V01–Y89).

Table 2 shows HRs corresponding to quintiles of ΔWC, Δweight and ΔHC for all-cause mortality from two models: model 1, a minimally adjusted model, includes age (as the underlying time variable), sex and country of birth, and model 2 includes age, sex, country of birth and the confounders identified from the causal diagram (Figure S1). The estimates changed minimally from model 1 to model 2, thus, the estimates from model 2 will be discussed. Participants who lost WC, weight or HC had higher all-cause mortality (HR: 1.26; 95% confidence interval (CI) 1.09–1.47 for WC; 1.80; 1.54–2.11 for weight; and 1.35; 1.15–1.57 for HC) than did those who had minimal changes. Increases in weight, WC or HC were not associated with all-cause mortality. The associations with all-cause mortality for change in WC and weight showed evidence of departures from linearity (likelihood ratio test for ΔWC, p-value = 0.03, Δweight, p-value≤0.001, and ΔHC, p-value = 0.08). For consistency with previously published studies, we also looked at the HRs for mortality associated with four groups of body size change representing decrease (<−3 units), stable (±3units), moderate gain (>3 and ≤10units) and extreme gain (>10units) of body size (Table S3); the results show similar associations to the results presented in Table 2.

**Table 2:** Hazard Ratios (95% Confidence Interval) for all-cause mortality in relation to change in body size.

				Model 1^a^			Model 2^b^	
	Deaths	Person-years	HR	95% CI	p-value	HR	95% CI	p-value
**Change in waist circumference (cm)**								
(−14.5 to 1.0]	438	34,147	1.32	[1.14, 1.53]	< 0.001	1.26	[1.09, 1.47]	0.002
(1.0 to 5.0]	301	33,980	1.00	-	-	1.00	-	-
(5.0 to 8.5]	257	31,756	0.99	[0.84, 1.17]	0.879	0.98	[0.83, 1.16]	0.793
(8.5 to 13.0]	260	34,416	0.99	[0.84, 1.17]	0.925	0.97	[0.82, 1.15]	0.764
(13.0 to 32.5]	209	30,738	1.10	[0.92, 1.31]	0.305	1.04	[0.87, 1.24]	0.700
**Change in weight (kg)**								
(−17.8 to −2.3 ]	595	31,833	1.88	[1.61, 2.21]	< 0.001	1.80	[1.54, 2.11]	< 0.001
(−2.3 to 0.7 ]	303	33,679	1.18	[0.99, 1.41]	0.059	1.18	[0.99, 1.41]	0.066
(0.7 to 3.3 ]	212	33,233	1.00	-	-	1.00	-	-
(3.3 to 6.6 ]	201	33,224	1.11	[0.92, 1.35]	0.271	1.08	[0.89, 1.31]	0.450
(6.6 to 24.6 ]	154	33,069	1.15	[0.93, 1.42]	0.190	1.01	[0.82, 1.25]	0.893
**Change in hips circumference (cm)**								
(−15.0 to −1.2 ]	423	32,936	1.39	[1.20, 1.62]	< 0.001	1.35	[1.15, 1.57]	< 0.001
(−1.2 to 1.8 ]	317	33,070	1.09	[0.93, 1.29]	0.279	1.08	[0.91, 1.27]	0.381
(1.8 to 4.5 ]	270	34,339	1.00	-	-	1.00	-	-
(4.5 to 8.0 ]	247	33,227	0.99	[0.84, 1.18]	0.950	0.96	[0.81, 1.15]	0.684
(8.0 to 25.8 ]	208	31,465	1.02	[0.85, 1.23]	0.816	0.97	[0.81, 1.16]	0.743

a Model 1: Estimates adjusted for sex and country of birth.

b Model 2: Estimates adjusted for sex, country of birth, quintile of socioeconomic status, body size at baseline, cumulative smoking status, and the following covariates measured at baseline and wave 2: physical activity, Mediterranean diet score and living alone.

### Cause-specific mortality

Participants who lost WC, weight, or HC had increased risk of CVD (WC: 1.39; 0.99–1.97, weight: 2.40; 1.57–3.65, and HC: 1.60; 1.09–2.35) but not obesity-related cancer mortality (Table 3). Increase in body size was not associated with increased CVD or obesity-related cancer mortality.

**Table 3:** Hazard Ratios (95% Confidence Interval) for death due to obesity-related cancer or CVD in relation to change in body size.

				Obesity cancer	related Deaths^a^^,^ b			CVD	deaths^a^
	Person-years	Deaths	HR	95% CI	p-value	Deaths	HR	95% CI	p-value
**Change in waist circumference (cm)**									
(−14.5 to 1.0]	25,962	30	1.27	(0.73, 2.23)	0.396	87	1.39	(0.99, 1.97)	0.059
(1.0 to 5.0]	25,775	21	1.00	-	-	53	1.00	-	-
(5.0 to 8.5]	24,022	22	1.19	(0.66, 2.16)	0.565	38	0.84	(0.55, 1.27)	0.408
(8.5 to 13.0]	25,946	16	0.87	(0.45, 1.66)	0.664	38	0.86	(0.57, 1.30)	0.469
(13.0 to 32.5]	23,036	20	1.41	(0.75, 2.64)	0.281	26	0.84	(0.53, 1.35)	0.476
**Change in weight (kg)**									
(−17.8 to −2.3]	24,268	37	1.20	(0.71, 2.02)	0.502	110	2.40	(1.57, 3.65)	≤ 0.001
(−2.3 to 0.7]	25,551	16	0.60	(0.32, 1.15)	0.123	55	1.59	(1.01, 2.52)	0.047
(0.7 to 3.3]	25,076	23	1.00	-	-	27	1.00	-	-
(3.3 to 6.6]	25,054	16	0.76	(0.40, 1.44)	0.404	34	1.49	(0.90, 2.47)	0.123
(6.6 to 24.6]	24,794	17	0.93	(0.49, 1.77)	0.836	16	0.99	(0.53, 1.85)	0.977
**Change in hips circumference (cm)**									
(−15.0 to −1.2]	24,992	31	1.19	(0.69, 2.05)	0.523	79	1.60	(1.09, 2.35)	0.017
(−1.2 to 1.8]	25,027	22	0.87	(0.49, 1.56)	0.651	59	1.27	(0.85, 1.89)	0.246
(1.8 to 4.5]	25,956	24	1.00	-	-	41	1.00	-	-
(4.5 to 8.0]	25,086	19	0.85	(0.47, 1.55)	0.599	34	0.91	(0.58, 1.44)	0.685
(8.0 to 25.8]	23,680	13	0.70	(0.35, 1.37)	0.296	29	0.96	(0.60, 1.56)	0.882

a Estimates adjusted for sex, country of birth, quintile of socioeconomic status, body size at baseline, cumulative smoking status, and the following covariates measured at baseline and wave 2: physical activity, Mediterranean diet score and living alone.

b Obesity-related cancers include the following cancers: breast, colorectal, endometrial, oesophageal, kidney, pancreatic.

### Sensitivity analyses

Age at baseline moderately modified the association between the change in body size and mortality (p-value from likelihood ratio test = 0.04, 0.04, 0.11 for WC, weight and HC, respectively). Older participants whose WC decreased had elevated HR, but younger participants did not (Table 4). The association with weight loss was stronger for older participants, whereas there was little difference in HR for decrease in HC according to age. In neither age group were increases in any measures associated with mortality.

**Table 4:** Hazard Ratios (95% Confidence Interval) for all-cause mortality in relation to change in body size by age group at baseline.

			< 55 years				≥ 55 years	
	Deaths (Person-years)	HR^a^	95% CI	p-value	Deaths (Person-years)	HR^a^	95% CI	p-value
**Change in waist circumference (cm)**								
(−14.5 to 1.0]	57 (17 258)	1.10	[0.75, 1.62]	0.620	381 (16 890)	1.31	[1.12, 1.54]	0.001
(1.0 to 5.0]	49 (17 841)	1.00	-	-	252 (16 139)	1.00	-	-
(5.0 to 8.5]	53 (17 936)	1.12	[0.76, 1.65]	0.583	204 (13 820)	0.95	[0.79, 1.14]	0.584
(8.5 to 13.0]	53 (20 170)	0.97	[0.65, 1.43]	0.865	207 (14 246)	0.98	[0.81, 1.18]	0.821
(13.0 to 32.5]	51 (19 849)	0.94	[0.63, 1.40]	0.762	158 (10 889)	1.06	[0.87, 1.30]	0.549
**Change in weight (kg)**								
(−17.8 to −2.3]	54 (11 786)	1.30	[0.88, 1.92]	0.189	541 (20 046)	1.92	[1.61, 2.30]	< 0.001
(−2.3 to 0.7]	48 (16 396)	1.05	[0.71, 1.56]	0.816	255 (17 282)	1.22	[1.00, 1.48]	0.053
(0.7 to 3.3]	51 (19 020)	1.00	-	-	161 (14 213)	1.00	-	-
(3.3 to 6.6]	57 (21 267)	0.99	[0.68, 1.45]	0.961	144 (11 957)	1.09	[0.87, 1.37]	0.438
(6.6 to 24.6]	53 (24 584)	0.73	[0.50, 1.08]	0.115	101 (8485)	1.16	[0.90, 1.49]	0.257
**Change in hips circumference (cm)**								
(−15.0 to −1.2]	60 (16 264)	1.44	[0.96, 2.13]	0.075	363 (16 672)	1.33	[1.13, 1.58]	0.001
(−1.2 to 1.8]	66 (18 017)	1.63	[1.11, 2.39]	0.013	251 (15 053)	0.98	[0.82, 1.18]	0.851
(1.8 to 4.5]	43 (19 558)	1.00	-	-	227 (14 781)	1.00	-	-
(4.5 to 8.0]	45 (19 731)	1.04	[0.68, 1.57]	0.871	202 (13 496)	0.95	[0.79, 1.15]	0.608
(8.0 to 25.8]	49 (19 484)	1.12	[0.74, 1.70]	0.579	159 (11 981)	0.93	[0.76, 1.14]	0.477

a Estimates adjusted for sex, country of birth, quintile of socioeconomic status, body size at baseline, cumulative smoking status, and the following covariates measured at baseline and wave 2: physical activity, Mediterranean diet score and living alone.

Sex, country of birth, baseline value of body size, smoking status, length of follow-up, self-reported health status, and diagnosis of previous disease did not modify the associations for all-cause mortality (results not shown).

## Discussion

In this cohort of middle-aged men and women, we found that decrease in body size, measured prospectively by WC, weight, or HC, was associated with an increased risk of all-cause mortality, particularly for older adults. Increase in body size was not associated with an increased risk of mortality.

The strengths of our study include its prospective design, almost complete follow-up of participants (only 12 (0.03%) participants were known to have left Australia), updated covariate information at wave 2, and direct measurements of body size, using standard protocols, at both waves.

The principal limitations of our study are (1) the small number of cause-specific deaths (i.e. deaths due to obesity-related cancer or CVD), which may explain why we did not observe an association between change in body size and obesity-related cancer mortality; (2) approximately 30% of participants alive at wave 2 did not attend the follow-up wave; and (3) the lack of information on intentionality of weight loss for the study participants.

The proportion of participants who were alive and attended wave 2 (i.e. 71.5%) was similar to that reported by others [10]. Participants who attended both waves were younger, better educated, and had a healthier lifestyle than non-participants, which may restrict the generalisation of our findings to populations of fairly healthy middle-aged adults. We previously conducted an extensive simulation study that showed that in the framework of this study, complete-case analysis provides unbiased estimates when compared to multiple imputation [27].

To attempt to overcome the criticism that the association between weight loss and increased mortality is due to inadequate control for confounding [28], we adjusted for several variables that might be associated with weight loss. For example, unintentional weight loss might reflect underlying diseases (reverse causation) or lifestyle characteristics (e.g. disability, cancer or respiratory disease) that lead to increased mortality [9], [29]. Further, our results were not sensitive to exclusion of participants diagnosed with cancer before wave 2, to whether participants had a history of disease (i.e. angina, diabetes or heart attack) diagnosed before wave 2, and self-rated health status at wave 2. Reverse causation as an explanation for the association between weight loss and mortality has been questioned [30].

A review of cohort studies showed that even when analyses were restricted to intentional weight loss, associations with mortality were inconsistent [31]. Large-scale randomised trials of weight loss with mortality as the outcome are the best way to provide the evidence, but these are expensive, would take many years and might not be feasible.

Despite our findings, obesity is associated with serious co-morbidities (e.g. impaired mobility, impaired quality of life, functional decline, glucose intolerance and increased risk of some cancers) and weight loss by obese individuals is associated with improved metabolic outcomes, reduced coronary heart disease and type 2 diabetes and a potential survival advantage [32]–[34].

We observed a stronger association for weight loss and mortality than for decrease in waist or hips circumference. Older, but not younger participants, at wave 2 who had lost weight had increased mortality. There are two possible explanations for this finding. First, WC and HC are more prone to measurement error than weight, and perhaps, more sophisticated methods of measuring lean mass and fat mass are necessary (i.e. information obtained from biomarkers, such as adipokines). However, these were not available for the second follow-up wave of this cohort. On the other hand, weight loss is associated with loss of both lean mass and fat mass, whereas WC is associated with loss of fat mass. Therefore, the stronger association between weight loss and mortality might be driven by the association with lean mass, which is consistent with loss of lean mass being associated with increased mortality [25]. This is consistent with age modifying the effect of weight loss on mortality, which may be a result of sarcopenia. Sarcopenia is the reduction of lean body mass with increasing age and is consistent with our finding that a third (35%) of participants lost weight from baseline to wave 2, whereas only 16% decreased their WC. Sarcopenia is associated with reduced physical activity, poor endurance, physical inactivity, inadequate nutrition, low gait speed and decreased mobility. As well, it has been associated with increased morbidities (e.g. congestive heart failure, chronic obstructive pulmonary disease, and type 2 diabetes) and has been independently associated with mortality in octogenarians, after adjusting for age and other relevant confounders [35], [36].

Results for the association between weight gain and all-cause mortality from observational cohort studies are inconsistent. Consistent with our findings, five studies reported no increased risk of mortality for an increase in body size [10], [12], [37]–[39]. Sauvaget et al. [40] found a decreased risk of mortality associated with moderate weight gain of 4–10%. Of the studies that reported an increased risk of mortality associated with weight gain, Bamia et al. [41] found a positive association for overweight and obese participants only, whereas Breeze et al. [42] found that participants who gained 10 kg or more between the two waves of data collection, measured 30 years apart, had a 1.4 fold increased risk of all-cause mortality (95% CI: 1.1–1.7). Somes et al. [43] analysed data collected for a clinical trial of antihypertensive drug treatment and found that a weight gain of more than 0.5 kg per year was associated with 2.4 fold increased risk of all-cause mortality (95% CI: 1.66–3.50). The participants' weight was measured quarterly over 4.5 years, with weight change representing a trend in weight based on a line of best fit through multiple weight measurements.

Perhaps alternative analyses are needed for observational cohort studies (e.g. causal modelling) or more frequent measures of body composition are necessary to identify the critical times for gaining or losing weight and the subsequent mortality risk. Further, as stated above, randomised control trials might be the best study design to answer this question, however this design is not without its limitations.

Our results were similar when follow-up was split at three years post wave 2. However, adverse consequences of weight gain may not manifest in the short-term [41], and three years of follow-up might not be sufficient to eliminate reverse causation since some conditions (for example chronic obstructive pulmonary disease and congestive heart failure) may remain undiagnosed for many years and cause weight loss or prevent typical weight gain [44].

In conclusion, our study provides further evidence that weight loss in mid to later life increases the risk of mortality. We recommend further investigation of this association in studies with longer follow-up and information about intentionality of weight loss. Further studies are also needed to understand the mechanisms underlying changes in fat and lean mass in older adults and their contributions to mortality. Current recommendations, based on observational cohort studies, should point towards healthy diet and physical activity for the prevention of weight gain into adulthood and weight stability from midlife to older age.

## Supporting Information

Figure S1 Causal diagram used to select confounding variables in the analysis.
(TIFF)

Table S1 Distribution of the baseline demographic and anthropometric characteristics of the MCCS participants.
(PDF)

Table S2 Spearman rank correlations between body size measured at baseline and wave 2 and change in body size in the Melbourne Collaborative Cohort Study.
(PDF)

Table S3 Hazard ratios (95% confidence interval) for all-cause mortality in relation to change in body size grouped in four categories.
(PDF)
