# Supplementary material for: Change in Body Size and Mortality: Results from the Melbourne Collaborative Cohort Study
Source: PLoS One. 2014 Jul 2;9(7):e99672. doi: 10.1371/journal.pone.0099672 (PMC4079561; doi:10.1371/journal.pone.0099672)
Supplement: Table S1 — Distribution of the baseline demographic and anthropometric characteristics of the MCCS participants. (PDF) [file pone.0099672.s002.pdf]

Table S1. Distribution of baseline demographic and anthropometric characteristics of the MCCS participants

		Wave 2 attendance		
			Did not attend	
	Baseline	Attended	Alive	Died
	n (%)	n (%)	n (%)	n (%)
**Participants**	41 514 (100.0)	26 984 (65.0)	10 732 (25.9)	3798 (9.1)
**Sex**				
Male	17 045 (41.1)	10 646 (39.5)	4218 (39.3)	2181 (57.4)
Female	24 469 (58.9)	16 338 (60.5)	6514 (60.7)	1617 (42.6)
**Age (years)**				
<50	13 152 (31.7)	9400 (34.8)	3465 (32.3)	287 (7.6)
50–59	13 507 (32.5)	9176 (34.0)	3509 (32.7)	822 (21.6)
≥60	14 855 (35.8)	8408 (31.2)	3758 (35.0)	2689 (70.8)
**Country of birth**				
Australia/New Zealand/UK	31 558 (76.0)	22 048 (81.7)	6729 (62.7)	2781 (73.2)
Southern Europe	9956 (24.0)	4936 (18.3)	4003 (37.3)	1017 (26.8)
**Highest level of education**				
Less than primary school	8046 (19.4)	3774 (14.0)	3273 (30.5)	999 (26.3)
Some high/technical school	15 853 (38.2)	10 218 (37.9)	4125 (38.5)	1510 (39.8)
Completed high/technical school	8576 (20.7)	5826 (21.6)	1962 (18.3)	788 (20.8)
Degree/diploma	9030 (21.8)	7164 (26.6)	1368 (12.8)	498 (13.1)
**Living alone**				
Not living alone	35 456 (85.4)	23 119 (85.7)	9260 (86.3)	3077 (81.1)
Living alone	6049 (14.6)	3863 (14.3)	1468 (13.7)	718 (18.9)

cont.

Table S1. Distribution of baseline demographic and anthropometric characteristics of the MCCS participants

		Wave 2	attendance	
			Did not	attend
	Baseline	Attended	Alive	Died
	n (%)	n (%)	n (%)	n (%)
**Alcohol intake**[1]				
Lifetime abstainers	11 871 (28.6)	7005 (26.0)	3766 (35.1)	1100 (29.1)
Ex-drinkers	1663 (4.0)	963 (3.6)	462 (4.3)	238 (6.3)
Low intake	22 515 (54.3)	15 387 (57.0)	5271 (49.2)	1857 (49.1)
Moderate intake	3599 (8.7)	2507 (9.3)	735 (6.9)	357 (9.4)
High intake	1827 (4.4)	1113 (4.1)	484 (4.5)	230 (6.1)
**Mediterranean diet score**				
≤3	9797 (23.6)	6097 (22.6)	2662 (24.8)	1038 (27.4)
4	8725 (21.0)	5607 (20.8)	2307 (21.5)	811 (21.4)
5	9624 (23.2)	6286 (23.3)	2498 (23.3)	840 (22.2)
6	7887 (19.0)	5275 (19.6)	1947 (18.2)	665 (17.6)
≥7	5435 (13.1)	3708 (13.7)	1299 (12.1)	428 (11.3)
**Physical activity score**				
None - 0	9223 (22.2)	5529 (20.5)	2800 (26.1)	894 (23.6)
Low - >0 & <4	8326 (20.1)	5458 (20.2)	2167 (20.2)	701 (18.5)
Moderate - ≥4 & <6	14 776 (35.6)	9398 (34.8)	3804 (35.5)	1574 (41.5)
High - ≥6	9180 (22.1)	6597 (24.4)	1957 (18.2)	626 (16.5)
**Smoking status**				
Never smokers	23 819 (57.4)	16 212 (60.1)	5990 (55.8)	1617 (42.6)
Former smoker	12 997 (31.3)	8328 (30.9)	3163 (29.5)	1506 (39.7)
Current smoker	4688 (11.3)	2441 (9.0)	1575 (14.7)	672 (17.7)

cont.

[1] Categories of alcohol intake are in grams/day as follows:
Low: Males: 1-39; Females: 1-19
Medium: Males: 40-59; Females: 20-39
High: Males: 60+; Females: 40+

Table S1. Distribution of baseline demographic and anthropometric characteristics of the MCCS participants

		Wave 2	attendance	
			Did not	attend
	Baseline	Attended	Alive	Died
	n (%)	n (%)	n (%)	n (%)
**Marital status**				
Married	28 500 (71.8)	18 718 (71.9)	7269 (71.9)	2513 (70.4)
Single	3615 (9.1)	2444 (9.4)	849 (8.4)	322 (9.0)
Divorced	3114 (7.8)	2056 (7.9)	826 (8.2)	232 (6.5)
de Facto	747 (1.9)	560 (2.2)	164 (1.6)	23 (0.6)
Widow	2801 (7.1)	1666 (6.4)	731 (7.2)	404 (11.3)
Separated	914 (2.3	576 (2.2)	264 (2.6)	74 (2.1)
**Index of Relative Socioeconomic Disadvantage**				
1st Quintile (most disadvantaged)	6083 (14.7)	3476 (12.9)	1917 (17.9)	690 (18.2)
2nd Quintile	8620 (20.8)	5014 (18.6)	2654 (24.8)	952 (25.2)
3rd Quintile	7668 (18.5)	4801 (17.8)	2116 (19.8)	751 (19.9)
4th Quintile	8447 (20.4)	5818 (21.6)	1975 (18.5)	654 (17.3)
5th Quintile (least disadvantaged)	10 559 (25.5)	7798 (29.0)	2025 (18.9)	736 (19.5)
