# Supplementary material for: Change in Body Size and Mortality: Results from the Melbourne Collaborative Cohort Study
Source: PLoS One. 2014 Jul 2;9(7):e99672. doi: 10.1371/journal.pone.0099672 (PMC4079561; doi:10.1371/journal.pone.0099672)
Supplement: Table S2 — Spearman rank correlations between body size measured at baseline and wave 2 and change in body size in the Melbourne Collaborative Cohort Study. (PDF) [file pone.0099672.s003.pdf]

Table S2. Spearman rank correlations between body size measured at baseline and wave 2 and change in body size in the Melbourne Collaborative Cohort Study

		Baseline			Wave 2			Change		
		Waist (cm)	Weight (kg)	Hips (cm)	Waist (cm)	Weight (kg)	Hips (cm)	Waist (cm)	Weight (kg)	Hips (cm)
Baseline	Waist (cm)	1.00								
	Weight (kg)	0.85	1.00							
	Hips (cm)	0.65	0.70	1.00						
Wave 2	Waist (cm)	0.82	0.80	0.60	1.00					
	Weight (kg)	0.75	0.91	0.64	0.86	1.00				
	Hips (cm)	0.50	0.62	0.76	0.71	0.74	1.00			
Change	Waist (cm)	-0.23	-0.03	-0.04	-	-	-	1.00		
	Weight (kg)	-0.09	-0.05	-0.01	-	-	-	0.64	1.00	
	Hips (cm)	-0.12	-0.01	-0.20	-	-	-	0.65	0.62	1.00
