# Supplementary material for: Change in Body Size and Mortality: Results from the Melbourne Collaborative Cohort Study
Source: PLoS One. 2014 Jul 2;9(7):e99672. doi: 10.1371/journal.pone.0099672 (PMC4079561; doi:10.1371/journal.pone.0099672)
Supplement: Table S3 — Hazard ratios (95% confidence interval) for all-cause mortality in relation to change in body size grouped in four categories. (PDF) [file pone.0099672.s004.pdf]

Table S3. Hazard ratios (95% confidence interval) for all-cause mortality in relation to change in body size grouped in 4 categories

					Model 1[a]			Model 2[b]	
	Deaths	Person-years	Rate[c]	HR	95% CI	p-value	HR	95% CI	p-value
**Change in waist circumference (cm)**									
**Change in waist circumference (cm)**									
(-14.5 to -3.0]	204	13,495	15.12	1.33	[1.12, 1.58]	0.001	1.25	[1.05, 1.48]	0.012
(-3.0 to 3.0]	377	36,210	10.41	1.00	-	-	1.00	-	-
(3.0 to 10.0]	517	63,437	8.15	0.90	[0.79, 1.02]	0.109	0.89	[0.78, 1.02]	0.096
(10.0 to 32.5]	367	51,895	7.07	0.94	[0.82, 1.09]	0.437	0.91	[0.79, 1.06]	0.223
**Change in weight (kg)**									
(-17.8 to -3.0]	524	25,890	20.24	1.77	[1.57, 1.99]	<0.001	1.68	[1.48, 1.89]	<0.001
(-3.0 to 3.0]	572	69,735	8.20	1.00	-	-	1.00	-	-
(3.0 to 10.0]	312	54,777	5.70	0.98	[0.85, 1.13]	0.799	0.94	[0.81, 1.08]	0.359
(10.0 to 24.6]	57	14,636	3.89	0.98	[0.74, 1.29]	0.874	0.82	[0.62, 1.09]	0.168
**Change in hips circumference (cm)**									
(-15.0 to -3.0]	294	20,495	14.35	1.43	[1.24, 1.65]	<0.001	1.40	[1.21, 1.61]	<0.001
(-3.0 to 3.0]	577	62,946	9.17	1.00	-	-	1.00	-	-
(3.0 to 10.0]	457	61,875	7.39	0.94	[0.83, 1.06]	0.298	0.93	[0.82, 1.05]	0.248
(10.1 to 25.8]	137	19,722	6.95	1.02	[0.84, 1.23]	0.855	0.98	[0.81, 1.18]	0.807

[a] Model 1: Estimates adjusted for sex and country of birth.
[b] Model 2: Estimates adjusted for sex, country of birth, quintile of socioeconomic status, body size at baseline, cumulative smoking status, and the following covariates measured at baseline and wave 2: physical activity, Mediterranean diet score and whether or not the participant lived alone.
[c] Rate per 1,000 person-years
